# Mapping Selenium Nanoparticles Distribution Inside Cells through Confocal Raman Microspectroscopy

**DOI:** 10.1021/acsami.5c00380

**Published:** 2025-03-18

**Authors:** Davide Redolfi-Bristol, Kenta Yamamoto, Wenliang Zhu, Osam Mazda, Pietro Riello, Elia Marin, Giuseppe Pezzotti

**Affiliations:** † Ceramic Physics Laboratory, Kyoto Institute of Technology, Sakyo-ku, Matsugasaki, Kyoto 606-8585, Japan; ‡ Dipartimento di Scienze Molecolari e Nanosistemi, Università Ca’ Foscari di Venezia, Via Torino 155, 30172 Venezia, Italia; § Department of Immunology, Graduate School of Medical Science, Kyoto Prefectural University of Medicine, 465 Kajii-cho, Kamigyo-ku, Kyoto 602-8566, Japan; ∥ Biomaterials Engineering Laboratory, Kyoto Institute of Technology, Sakyo-ku, Matsugasaki, Kyoto 606-8585, Japan; ⊥ Department Polytechnic of Engineering and Architecture, University of Udine, 33100 Udine, Italy; # Biomedical Research Center, Kyoto Institute of Technology, Sakyo-ku, Matsugasaki, Kyoto 606-8585, Japan; ∇ Biomedical Engineering Center, Kansai Medical University, 1-9-11 Shinmachi, Hirakata, Osaka 573-1191, Japan; ○ Department of Dental Medicine, Graduate School of Medical Science, Kyoto Prefectural University of Medicine, 465 Kajii-cho, Kamigyo-ku, Kyoto 602-8566, Japan; ◆ Department of Orthopedic Surgery, Tokyo Medical University, 6-7-1 Nishi-Shinjuku, Shinjuku-ku, Tokyo 160-0023, Japan; ¶ Department of Applied Science and Technology, Politecnico di Torino, Corso Duca degli Abruzzi 24, 10129 Torino, Italy

**Keywords:** selenium nanoparticles, cytotoxicity, Raman microspectroscopy, 2D mapping, 3D imaging

## Abstract

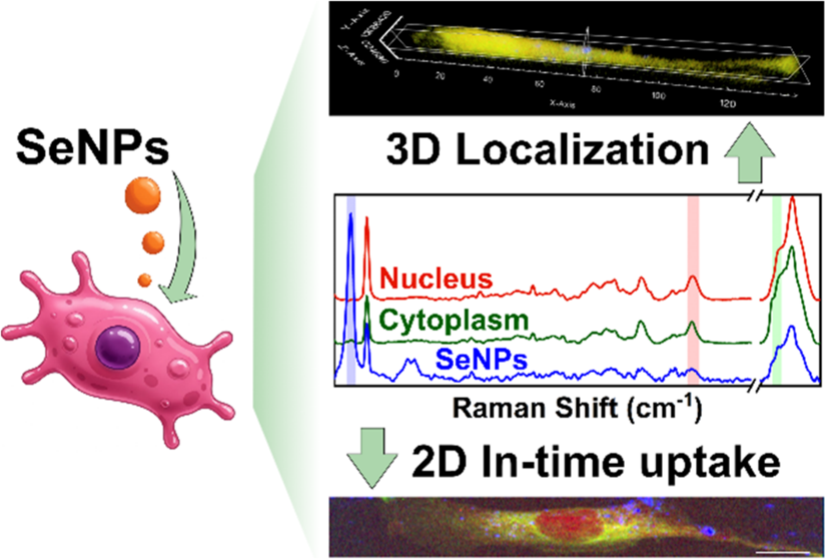

Selenium nanoparticles (SeNPs) exhibit significant potential in biomedical applications due to their antimicrobial, anticancer, and anti-inflammatory properties. In this study, we synthesized biocompatible SeNPs and employed confocal Raman microspectroscopy to map their distribution within human dermal fibroblast (HDF) cells. SeNPs possess a distinctive Raman band placed outside the cellular fingerprint region, which facilitates its detection and precise Raman imaging. Viability assays revealed that SeNPs exhibit cytotoxic effects only at the highest concentrations and for long exposure times while resulting in no harmful effects during all of the other treatments. For the first time, we achieved three-dimensional (3D) Raman mapping of SeNPs within cells, providing insights into their cellular penetration. Additionally, two-dimensional (2D) Raman mapping performed at different times and at sublethal concentrations demonstrated dynamic uptake and confirmed internalization. These findings highlight the effectiveness of SeNPs for biomedical imaging and therapeutic applications, offering an additional approach to studying nanoparticle–cell interactions.

## Introduction

Selenium nanoparticles (SeNPs) have recently gained large attention thanks to their antimicrobial, anticancer, and anti-inflammatory properties.^1,2^ One of the key advantages of SeNPs lies in their lower toxicity compared with other selenium compounds, such as selenite and selenate salts, as elemental selenium is recognized as the least toxic form of this essential trace element. Selenium is crucial for human health, but its therapeutic and toxic doses are narrowly separated.^3^ The United Kingdom group of vitamins and minerals recommends an intake of around 60 μg per day, with a threshold of 400 μg beyond which selenium toxicity may occur.^4^ In nanoparticle form, elemental selenium could therefore be the best option for biomedical applications. Indeed, studies have demonstrated the absence of SeNPs’ toxicity both *in vitro* and *in vivo*,^5,6^ while additionally showing the ability of SeNPs to mitigate oxidative stress in Alzheimer’s disease.^7^

Selenium nanoparticles can be produced through either chemical or biological methods and are relatively cost-effective due to the low price of their synthetic precursors.^1,2^ This economic advantage becomes particularly pronounced when compared to other metal nanoparticles, such as gold (AuNPs) and silver nanoparticles (AgNPs). Additionally, their synthesis through biological route, employing bacteria, fungi, or plant extracts, not only contributes to their economic viability but also aligns with environmentally friendly practices, making SeNPs an advanced and accessible option for various applications.^8−10^ For these reasons, SeNPs are generally considered as cheap, biocompatible, and antioxidant materials with promising uses in biomedical applications, such as drug delivery and imaging.^11−13^ Khalid et al. have prepared fluorescent SeNPs and applied them for cellular imaging with successful results.^13^ Nevertheless, the origin of the fluorescence has not been completely elucidated. In addition, the most intense fluorescence signal was acquired when the NPs were coated with a polymeric coating, which modified the refractive index of the system. Therefore, it is not certain whether in diverse conditions, such as different culture medium, capping agent, or surface functionalization, the fluorescence phenomenon could be reduced or completely hampered. Additionally, fluorescence microscopy cannot always be applied, especially in cases in which prolonged measurements must be performed, which could lead to photobleaching. Another limitation of fluorescence microscopy is that the use of a specific illumination source to excite one fluorescent NPs may preclude the simultaneous tracking of other NPs or molecules with overlapping excitation spectra. These limitations reduce the suitability of fluorescence microscopy for tracking nanoparticles, particularly when multiple types of NPs need to be distinguished and monitored simultaneously. Alternative methods for examining nanoparticle localization within cells include, for example, transmission electron microscopy (TEM) and X-ray microscopy (XRM). However, both techniques involve extensive sample preparation: TEM necessitates fixation with oxidative and toxic compounds and ultramicrotoming, and the NPs must exhibit adequate electronic contrast within the cellular environment, while XRM requires the use of contrast agents and access to synchrotron facilities.

Ideally, methods for localizing and recognizing internalized NPs within cells should rely on their inherent chemical composition as opposed to electronic contrast or the characteristics of external labels. In addition, uncovering the specific subcellular locations of NPs within the cytoplasm (such as endosomes, lysosomes, or the nucleus) could significantly enhance the comprehension of their intracellular trafficking and interaction mechanisms. A promising technique that could fulfill these requirements is confocal Raman microspectroscopy.^14−16^ Confocal Raman microspectroscopy is an analytical technique that combines the depth-resolving capability of confocal microscopy with the molecular specificity of Raman Microspectroscopy, allowing for three-dimensional imaging and providing detailed information about the chemical composition and spatial distribution of NPs inside the cells, in a label-free manner.^14,17^ It is advantageous over other techniques (such as fluorescence microscopy) since each peak of the Raman spectra is unique and attributable to a single molecule or group of molecules, minimizing the risk of signal confusion with other fluorescent elements that could be activated by the same illumination source. This specificity also ensures that nanomaterials are not mistaken for other components, as some NPs can exhibit distinct and identifiable Raman signals. These features, together with the distinctive combination of high spatial resolution, chemical specificity, minimal sample preparation, and nondestructive analysis make Raman microspectroscopy a valuable tool in biomedical application.^17−19^ Raman microspectroscopy has shown promising results for microbial identification,^20^ cell classification,^21^ and sorting^22^ together with studies about cellular processes like differentiation,^23,24^ activation,^25,26^ and death.^27,28^ Thanks to the specific vibrations of biological molecules, Raman microspectroscopy allows subcellular analysis, enabling the identification of specific components within cellular compartments such as the cytoplasm, nuclei, and even the cell membrane. Cellular tomographic reconstruction can therefore be performed, allowing three-dimensional visualization of individual cells.^29,30^ In this context, Raman spectroscopy has shown interesting results in the analysis of intracellular distribution of nanomaterials.^31−34^ NPs possess specific structural vibrations that can be observed through Raman spectroscopy; however, for most materials, these vibrations produce weak signals that are hindered by the background or cells’ signal, making them indetectable in cellular environment. A class of nanomaterials that can be easily mapped are metallic nanoparticles (*e.g.*, AuNPs, AgNPs) thanks to the occurrence of surface enhancement Raman scattering (SERS) phenomenon.^16^ Nevertheless, the large intensity enhancement caused by the SERS effect limits the observation of the other Raman signals, therefore preventing the understanding of the entire cellular molecular composition. Only a limited number of NP types, such as TiO_2_,^32^ MoS_2_,^33^ or polystyrene,^34^ have displayed distinct Raman vibrations with sufficient intensity to be distinguishable within cells. However, several studies report the cytotoxicity of these materials and, in addition, their precise localization inside cells required intricate calculations for image reconstruction, further complicating the generation of three-dimensional (3D) maps to prove their distribution and making the technique less user-friendly.^32,35^ In contrast, SeNPs appear to show biocompatible properties together with possessing a specific Raman signal located outside the typical Raman fingerprint region of cells. These characteristics make them ideal for Raman imaging purposes, yet their potential has never been explored.

In our work, we monitored for the first time the distribution of SeNPs inside human dermal fibroblast (HDF) cells using Confocal Raman Microspectroscopy. SeNPs have been easily produced through chemical reduction of selenium precursor in the presence of l-cysteine and tannic acid. The synthesis resulted in the formation of stable nanoparticles around 76 nm in diameter. Cytotoxicity of SeNPs has been tested against healthy HDF cells for short and long exposure times, resulting in a decrease in viability only for the highest concentration tested after 72 h. The localization of SeNPs inside the cells has been achieved through Confocal Raman Microspectroscopy, reconstructing the Raman images from the original Raman spectra without the use of complex calculations. From the Raman maps, it has been possible to confirm the NPs internalization, proved also by the 3D reconstruction of cells through Raman signals. Eventually, to confirm that these nanoparticles could be detected even at low and nontoxic concentrations, uptake dynamic and localization of SeNPs inside cells have been performed at different time points at a concentration 10 times lower than the harmful one. These results demonstrate how selenium nanoparticles could be a promising and innovative material for biomedical applications when coupled with the confocal Raman microspectroscopy technique.

## Materials and Methods

### Materials

Sodium selenite (Na_2_SeO_3_), l-cysteine, and tannic acid were purchased from Sigma-Aldrich (Merck KGaA, Germany). Dulbecco’s modified Eagle’s medium (DMEM), fetal bovine serum (FBS), MEM nonessential amino acid solution, l-sodium pyruvate, penicillin-streptomycin mixed solution, cell count reagent SF, and phosphate-buffered saline (PBS) solutions were purchased from Nacalai Tesque (Japan).

### Synthesis of Selenium Nanoparticles (SeNPs)

9.75 mL of distilled water, 0.25 mL of Na_2_SeO_3_ (0.1 M), and 0.1 mL of tannic acid (10 mM) were inserted in a flask under vigorous stirring. After 5 min of homogenization, 2 mL of l-cysteine (0.05 M) was slowly dropped in the solution. The reaction is then left under stirring at room temperature for 30 min. After 30 min, the solution is centrifuged at 10,000 rpm for 15 min to remove the excess reagents and redispersed in distilled water. Eventually, the solution is stored at 4 °C.

### Characterization of SeNPs

#### Scanning Electron Microscopy (SEM)

Scanning electron microscopy (SEM) images were acquired using a Zeiss Sigma VP field emission scanning electron microscope (FE-SEM) equipped with an in-lens electron detector working in high-vacuum mode and an EHT voltage of 10 kV (Germany). Average SeNPs diameter was assessed by counting around 460 nanoparticles in different positions of the sample, for both the freshly prepared sample and the sample aged 10 months; ImageJ software was used for the analysis.^36^

#### Dynamic Light Scattering (DLS)

Dynamic light scattering (DLS) data of the hydrodynamic diameter of the nanoparticles were acquired using a Zetasizer Ultra instrument (Malvern Panalytical, Spectris, United Kingdom). Plastic cuvettes (DTS0012) were used, and measurements were performed in triplicate.

#### ζ-Potential

ζ-Potential measurements were performed using an ELSZ-1000 (Otsuka, Japan). The instrument combines dynamic light scattering (DLS) and ζ-potential measurements. For these experiments, 5 mL of nanoparticulate suspension was diluted with water. The sample was equilibrated for about 30 min, and then the ζ-potential was recorded.

#### Small-Angle X-ray Scattering (SAXS)

Small-angle X-ray scattering (SAXS) data were acquired using Malvern Panalytical equipment constituted of a diffractometer (Empyrean), a SAXS/WAXS chamber (ScatterX78), and a solid-state detector (PIXcel3D). The incoming slit collimated Cu Kα beam was focalized by an elliptically bent, one-dimensional (1D) graded, multilayer X-ray mirror; Cu Kβ contamination was less than 0.1%. Data were fitted using a polydisperse noninteracting Schultz distribution of spheres.

#### X-ray Diffraction (XRD)

X-ray powder diffraction patterns were recorded with conventional Bragg–Brentano geometry at 295 K, with a step size of 0.04°, on a scale of 10–80° 2θ and a time per step of 2000 s/step. An Empyrean diffractometer (Malvern Panalytical Ltd.) equipped with Bragg–Brentano HD incident optics, 1/8° divergence slit, and copper X-ray tube (wavelength Kα 1.5406 Å) at 40 kV and 40 mA was used. The detector used is a hybrid 2D solid-state pixel detector, PIXcel3D (255 active channels).

#### Raman Spectroscopy

Raman spectra of SeNPs were collected with a RAMANtouch spectroscope (Nanophoton Co., Osaka, Japan) depositing a drop of the colloidal solution on a CaF_2_ substrate and letting it dry at RT. The RAMANtouch spectroscope was operated in “point-mode”, using an excitation source of 532 nm (excitation power = 4.19 mW), a 300 gr/mm grating, and a 50× objective lens (NA = 0.8). Different spots of the sample were irradiated for 1 s and the acquisitions were averaged 4 times. The averaged spectrum underwent the following processing: background subtraction, smoothing (Savitsky-Golay smoothing; Degree 2, Size 7, Height 11), and baseline correction (manually selecting the points representative of the background) by means of LabSpec software (version 5.5, Horiba, Japan).

#### Transmission Electron Microscopy (TEM)

Transmission electron microscopy (TEM) images and diffraction patterns were acquired using a TEM Jeol JEM Cold FEG F200, operated at 200 kV. The sample was drop-cast on a holey carbon Cu copper grid for the analysis.

#### Photoluminescence Spectroscopy

Photoluminescence spectra have been acquired by irradiating a colloidal solution of SeNPs by an LED source with an excitation wavelength centered at 510 nm and detecting the signal at a window of 550–900 nm by means of a QEPro-XR spectrometer (Ocean Optics).

#### Inductively Coupled Plasma-Optical Emission Spectroscopy (ICP-OES)

For Se quantification, 1 mL of SeNPs solution was dissolved in a mixture of 8.5 mL of distilled water and 0.5 mL of freshly prepared aqua regia with ultrapure acids (HCl/HNO_3_ = 3:1). After 1 h, the samples were analyzed by means of inductively coupled plasma-optical emission spectroscopy (ICP-OES) PerkinElmer Optima 5300SV (PerkinElmer). Selenium (λ=196.026) has been quantified using an external five-point calibration curve ranging from 1.25 to 20 mg/L.

#### Analytical Centrifuge

LUMiSizer Dispersion Analyser (LUM GmbH, Germany) was used to measure the sedimentation velocity of the nanoparticles. LUM 2 mm, PC, Rect. Synthetic Cell (110–132xx) were used as cuvette and filled with 400 μL of SeNPs colloidal solution. Measurements have been performed in 6 copies for each sample.

### Cell Culture

Normal human dermal fibroblasts (HDF) derived from 22-year-old black females were purchased from Toyobo Life Science (Osaka, Japan). The HDF cells were cultured in Dulbecco’s modified Eagle’s medium (DMEM) containing phenol red, supplemented with 10% v/v fetal bovine serum (FBS), 1% MEM nonessential amino acid solution, 1% l-sodium pyruvate and 1% penicillin-streptomycin mixed solution (complete medium), in a humidified incubator at 37 °C and 5% CO_2_ conditions.

### Cell Viability Quantification

Cell viability quantification was performed by a WST-8 assay (Cell Count Reagent SF, Nacalai Tesque, Japan) based on the cleavage of tetrazolium salt by metabolically active cells to form a water-soluble formazan dye. Briefly, HDF cells were seeded in 24-well plates (2 × 10^4^ cells/well) and incubated overnight. SeNPs were added to the wells to final concentrations of 0.15, 1.5, and 15 μg/mL, and incubated for additional 24 and 72 h. At the end of the treatment, the medium from each well was removed, and the cells were washed one time with PBS to remove possible interference from SeNPs. Cells were incubated with 450 μL of complete culture media and 50 μL of WST-8 solution for 2 h in an incubator. Subsequently, two aliquots of 100 μL were taken from each well and placed in a 96-well plate. Eventually, absorbance at 450 nm was measured by an Infinite F50 Plus microplate reader (Tecan, Switzerland). The cell viability of each group is expressed as a percentage of the mean value of the control. The measurements were carried out in triplicate.

### Raman Microspectroscopy

For Raman studies, HDF cells were grown in complete growth medium in an incubator at 37 °C and 5% CO_2_ for 24 h on CaF_2_ dishes, to minimize background signal as reported in the literature. Subsequently, the cells were treated with 1.5 or 15 μg/mL of SeNPs diluted in supplemented DMEM medium for 4, 12, and 24 h. Prior to Raman microspectroscopy, the cells were fixed with formaldehyde solution. Briefly, the cells were washed twice with PBS, then treated with 4% formaldehyde solution for 5 min, and finally washed 3 times with PBS. Raman microspectroscopy measurements were performed while CaF_2_ dishes were kept in PBS.

Raman spectra of HDF cells were collected with a dedicated Raman device (RAMANtouch, Nanophoton Co., Osaka, Japan). The RAMANtouch spectroscope was operated with an excitation source of 532 nm (excitation power density = 3.7 mW/μm^2^), a 300 gr/mm grating, and a 60× immersion objective lens (NA = 1.0). The spectral resolution was about 3 cm^–1^ and was collected in the range from 180 to 3050 cm^–1^. The RAMANtouch spectroscope was operated in “line-mode”, acquiring up to 400 simultaneous spectra per line and performing maps of around 140 × 20 μm^2^ with a step size of 0.4 μm between each line. The exposure time for each line was 10 s and the acquisition was averaged one single time per line. The resulting acquisition time was around 10–20 min per map. All of the experiments were performed in triplicate, on at least three cells per replicate. Confocal Raman Maps were acquired with the same procedure but with few adjustments: HDF cells were treated with 15 μg/mL of SeNPs; the exposure time for each line was 5 s; the step size on the *z*-axis was 1 μm between each map, performing at least 9 steps and resulting in an acquisition time per cell of around 1 h. Raman maps were reconstructed using the “Area” mode of the RAMAN Viewer software (Nanophoton Co., Osaka, Japan), integrating the area below the peak of interest, and subtracting it from the areas close to the peak itself. More details about the data processing are reported in Figure S1. No additional spectral processing has been performed to reconstruct the maps.

To show the Raman spectra in the manuscript, Raman data were processed through RAMAN Viewer software averaging the total spectra in each area corresponding to “cytoplasm”, “nucleus”, “background” and “SeNPs” (Figure S2a). The raw averaged spectra of each area are reported in Figure S2b. The raw averaged spectrum underwent the following processing: background subtraction, smoothing (Savitsky-Golay smoothing; Degree 2, Size 7, Height 11), and baseline correction (manually selecting the points representative of the background) by means of LabSpec software (version 5.5, Horiba, Japan). Eventually, standard normal variate (SNV) normalization was performed using Origin software (Version 9.8.0.200, OriginLab, Massachusetts).

## Results and Discussion

**Figure 1:**
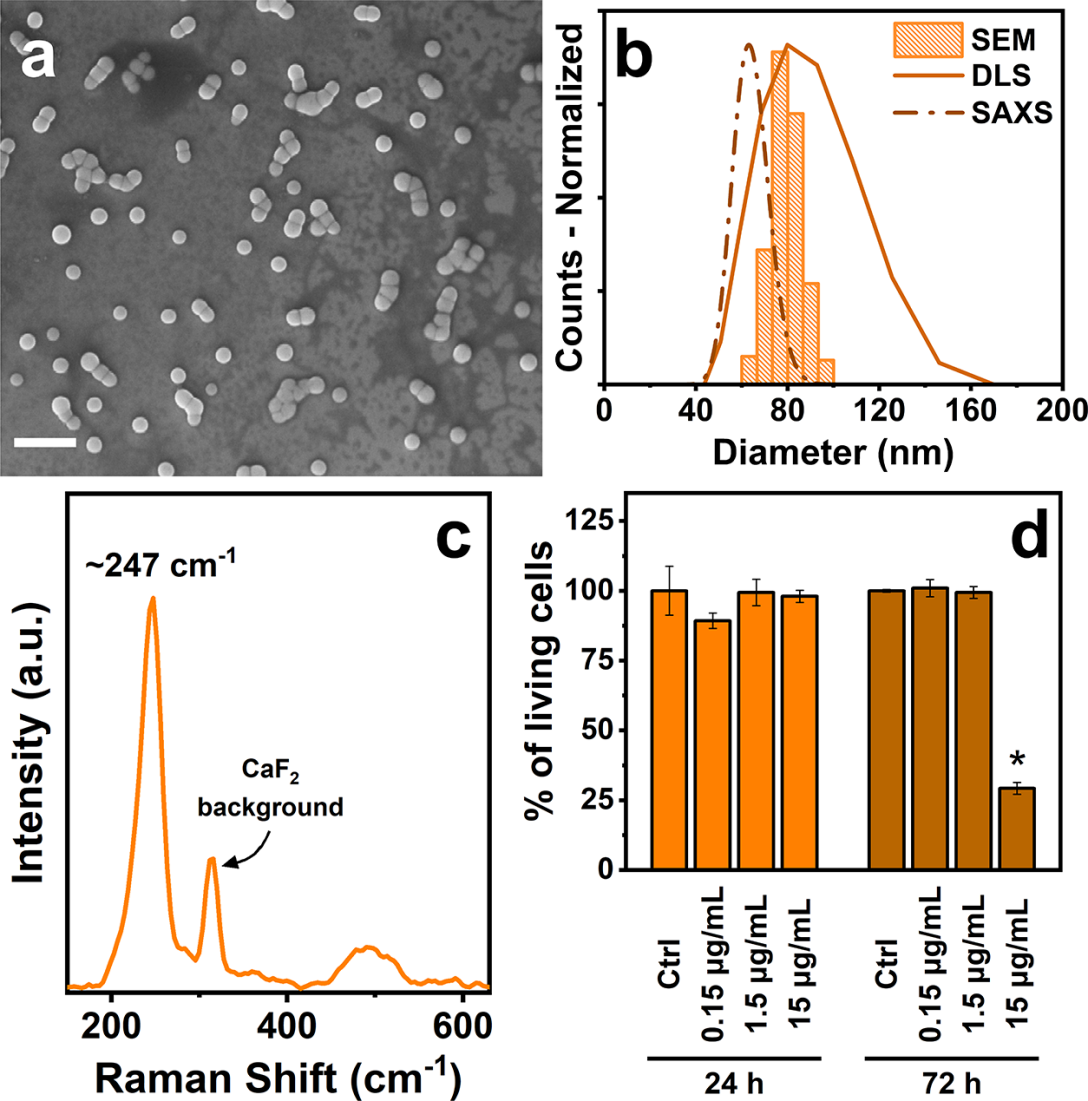
(a) SEM image of SeNPs (scale bar: 300 nm), (b) SEM, DLS, SAXS distributions, and (c) Raman spectra of SeNPs. (d) Quantification of living cells after incubation with SeNPs for 24 and 72 h. *Significant differences from negative control (*P* < 0.05).

Selenium nanoparticles have been easily synthesized through the reduction of Na_2_SeO_3_ by means of l-cysteine in the presence of tannic acid. After the addition of the reducing agent, the transparent mixture of sodium selenite and tannic acid turned quickly into an orange and turbid solution, confirming the formation of SeNPs. The obtained product was thoroughly characterized to assess its physicochemical properties. Figure 1a reports a representative SEM image of the selenium nanoparticles. The NPs show a spherical shape with a homogeneous distribution of 76 ± 7 nm, measured by SEM. Their dimension has additionally been confirmed through DLS and SAXS analysis, displaying sizes of 82 nm (PDI = 0.016) and 64 ± 8 nm, respectively, for the two techniques (Figure 1b). The small variations in size estimation between the methods could be caused by the solvation effects that the particles experience during the measurements. ζ-Potential of the SeNPs suspension has been measured resulting in a negative value of −55.4 mV, demonstrating colloidal stability by electrostatic repulsion of NPs in water. High stability in aqueous suspensions is particularly advantageous for biomedical applications, such as drug delivery or imaging as it ensures that NPs remain well dispersed and do not form aggregates that could affect their performance or biodistribution. To confirm the stability of the prepared SeNPs, SEM imaging, DLS, and ζ-potential analysis have been performed after 10 months on the same sample kept in a fridge at 4 °C. Initially, the colloidal solution presented a precipitate on the bottom of the vessel due to the deposition of the NPs during time. Nevertheless, after 5 min of sonication, the deposit redispersed and the solution returned orange. After 10 months, SeNPs still showed a spherical shape with only a slightly smaller average diameter of 73 ± 6 nm, measured by SEM (Figure S3). The hydrodynamic diameter measured by DLS displayed a size of 81 nm (PDI = 0.05), while ζ-potential was still highly negative (−57.8 mV), confirming the colloidal stability of the NPs in water for prolonged periods of time.

XRD analysis and Raman spectroscopy were carried out to study the crystalline nature of the nanoparticles. The diffractogram of SeNPs presents a broad signal around 28° (Figure S4a), while the Raman spectrum shows an intense peak at 247 cm^–1^ (Figures 1c and S4e). In the Raman spectrum, it is also possible to notice another weaker large peak around 500 cm^–1^, yet its origin has not been determined. Analysis of the raw spectra of SeNPs, l-cysteine, and tannic acid (Figure S4e) confirms that the peaks are exclusive to SeNPs, as they are absent in the spectra of the capping agents. The background observed in the SeNPs spectra is likely attributed to tannic acid on the nanoparticle surface, though it is significantly less intense compared to the prominent peak at 247 cm^–1^. XRD and Raman signals suggest that the structure of the NPs is amorphous,^37,38^ however there could be the possibility that they are constituted of small crystalline grains of rhombohedral Se_6_ ring structure.^39,40^ To elucidate this possibility, we decided to perform a high-resolution TEM analysis. Figure S4b–d presents TEM images and diffraction patterns of SeNPs which, however, do not exhibit any specific crystalline planes. This observation supports the hypothesized amorphous nature of the NPs. Nevertheless, it is important to note that recent studies have proven that even the use of an overly intense Raman laser can easily induce the transition of Se rings into chains.^41^ This phenomenon complicates the accurate attribution of the crystalline phase for this type of material, especially when TEM, which employs a source that is more powerful than a simple laser.

TEM images also reveal the presence of lighter regions within the SeNPs spheres (Figure S4b,c). These spots suggest that the nanoparticles may possess a porous structure. This characteristic could make them even more promising materials, enabling their potential extensive functionalization with therapeutic drugs for combined imaging and drug delivery applications. The most commonly employed techniques for quantifying material porosity include gas adsorption methods such as Brunauer–Emmett–Teller (BET) surface area analysis and the Barrett–Joyner–Halenda (BJH) method as well as mercury intrusion porosimetry and helium pycnometry. However, these methods typically require relatively large sample quantities (0.1–10 g) and are often limited in their ability to assess closed pores. Given that our SeNPs synthesis yields a maximum of approximately 1.5 mg per batch and that TEM analysis suggests the presence of pores, these conventional approaches are unsuitable for our study. To overcome these limitations, we quantified SeNPs porosity by first determining their apparent density (ρ_P_). This was achieved by measuring their sedimentation velocity (*v*) in solution using analytical centrifugation, providing an alternative method to assess porosity under our experimental constraints.^42−44^ Indeed, by applying Stokes’ law to describe sedimentation velocity in a centrifugal field (eq 1), the apparent density of the nanoparticles (ρ_P_) can be determined (eq 2)
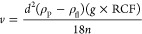
1
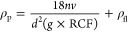
2where *d* is the average nanoparticle diameter measured by SEM; *n* and ρ_fl_ are the viscosity and density of water dispersion medium (*n* = 0.8991 mPa·s and ρ_fl_*=* 997.3 kg/m^3^), respectively; *g* is the gravitational acceleration; and RCF is the relative centrifugal force experienced by the nanoparticles. Furthermore, assuming that the nanopores are filled with the surrounding fluid since they have never been completely dried and that the solid component consists of amorphous selenium (ρ_S_ = 4280 kg/m^3^), the average porosity of the nanoparticles can be calculated using eq 3
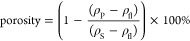
3

Table 1 summarizes the average sedimentation velocity obtained from the sedimentation velocity distribution (Figure S5) measured for four SeNPs samples of different sizes synthesized by varying the concentration of tannic acid in solution. This was done to confirm that, regardless of NPs size, the apparent density and porosity remained constant, thereby indicating that these properties are intrinsic to the selenium nanoparticles produced by our preparation method. The results indicate that the apparent density of SeNPs is consistently lower than that of bulk amorphous selenium, averaging around 3450 ± 150 kg/m^3^ regardless of the NPs size. Similarly, the porosity remains approximately 25 ± 5% across all samples. These findings confirm that the synthesized SeNPs possess a porous structure.

**Table 1:** Porosity Quantification Results for Different Types of SeNPs

tannic acid concentration (mM)	average SEM diameter (*d*) (nm)	sedimentation velocity (*v*) (μm/s)	RCF	apparent SeNPs density (ρ_P_) (kg/m^3^)	porosity (%)
0	157 ± 19	61 ± 9	2112	3281	30
5	116 ± 10	72 ± 13	2120	3519	23
20	135 ± 11	28 ± 5	2119	3619	20
50	93 ± 7	44 ± 8	2115	3519	23

Khalid et al. have reported a similar synthesis of SeNPs with a different reducing agent, showing the occurrence of a photoluminescent signal for their nanoparticles. To verify if our SeNPs could also exhibit this property, we decided to collect fluorescence spectra of the colloidal solution with an excitation wavelength of 510 nm and a detection window of 550–900 nm. Figure S6 reports the results of the measurement and shows the absence of any photoluminescent signal. This fact supports the hypothesis that SeNPs are not intrinsically photoluminescent and that their fluorescence property could maybe depend on the solution in which they are dispersed or the capping agent on their surface.

To verify the biocompatibility of SeNPs, a WST-8 assay was performed on human dermal fibroblast (HDF) cells after exposure to the nanoparticles for 24 and 72 h at different concentrations. Prior to performing cytotoxicity tests, the total selenium concentration in the solutions was quantified by ICP-OES in order to express the exposure concentrations in μg/mL and to evaluate the amount of selenium with which the cells interacted. To avoid misinterpretations, cells were washed once with PBS to remove possible nanoparticles deposited on the bottom before being treated with the WST-8. Furthermore, the same tests in the presence of only SeNPs dispersed in culture media were performed and showed the same absorbances as pure culture media, demonstrating the absence of alteration due to the possible presence of SeNPs remaining in wells. The results reported in Figure 1d show that SeNPs exhibit cytotoxic effects only at the highest concentration tested (15 μg/mL) and for the longest exposure times (72 h), while resulting in no harmful effects during all of the other treatments. Specifically, only cells treated with 15 μg/mL SeNPs for 72 h exhibited a viability reduction of approximately 75% compared with the untreated control. In all other cases, the observed variations in cell viability were within the range of statistical variability, suggesting that SeNPs can be considered nontoxic up to 1.5 μg/mL. Notably, the toxic concentration level observed in our study is lower than those reported in previous studies.^11,13,45^ This discrepancy may be attributed to the potential toxicity of the capping agents used or residual unreacted selenite salts that were not completely removed during purification. Additionally, the amorphous nature of SeNPs may be a factor influencing their toxicity. Literature reports conflicting findings, with some studies suggesting that crystalline SeNPs are less toxic than amorphous ones, while others indicate the opposite.^46−48^ To date, no comparative analysis has been conducted to precisely determine the toxicity differences between amorphous and crystalline SeNPs. The available literature on the toxicity of crystalline SeNPs is limited, and the nanoparticles investigated vary in size and surface functionalization, making accurate comparisons and toxicity attributions challenging. Furthermore, amorphous SeNPs have demonstrated higher bioavailability than their crystalline counterparts when exposed to bacteria, fungi, or plants, whereas crystalline SeNPs have exhibited stronger antimicrobial activity.^49,50^ These findings suggest that the irregular structure of amorphous SeNPs may facilitate their reduction or oxidation, leading to their disintegration. In contrast, the more stable crystalline phase may be less prone to degradation, potentially allowing it to persist longer in biological systems and exert prolonged effects. Further studies are required to comprehensively assess these possibilities and their implications.

Despite these findings, the concentrations and exposure times utilized in this study were adequate for subsequent experiments. Furthermore, since the Raman properties of SeNPs remain consistent regardless of the capping agent, substituting the current stabilizing agents with more biocompatible alternatives and improving the washing process could optimize the use of SeNPs for biomedical Raman imaging applications.

**Figure 2:**
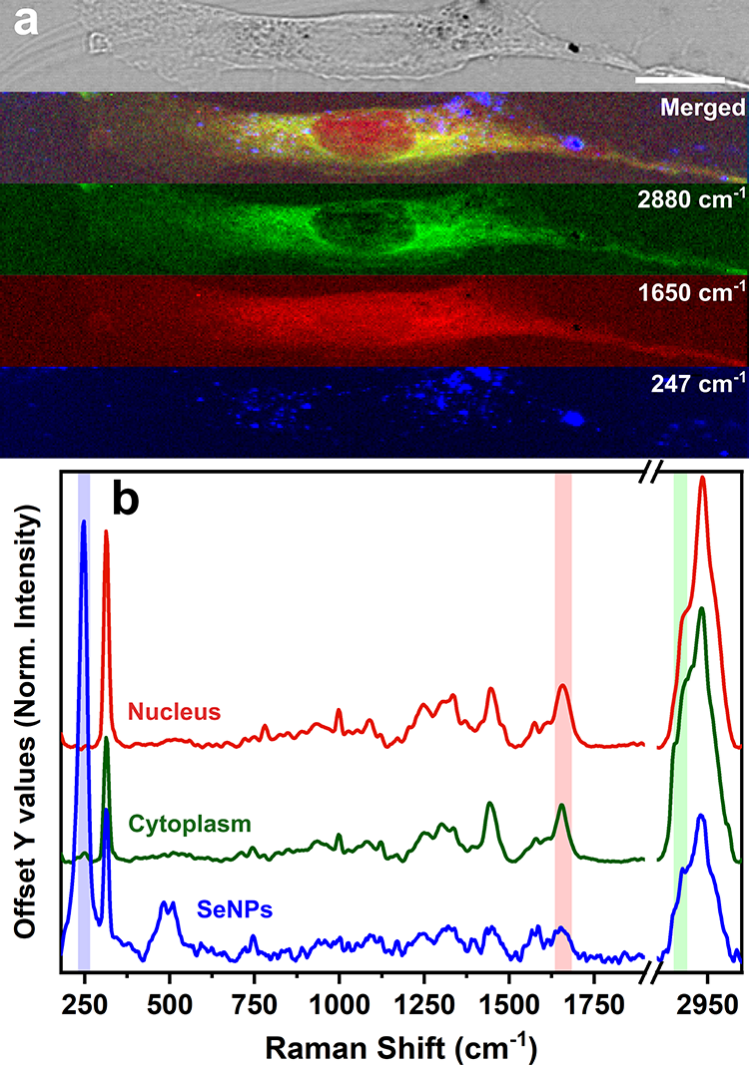
(a) Optical micrograph and Raman images obtained from signals at ∼2880 cm^–1^ (lipids), ∼1650 cm^–1^ (proteins), and ∼247 cm^–1^ (SeNPs) of HDF cells incubated with SeNPs at 15 μg/mL for 24 h (scale bar: 20 μm). (b) Average processed Raman spectra from the nucleus, cytoplasm, and internalized SeNPs.

Raman imaging has been performed to have a direct visualization of the SeNPs localization in fixed HDF cells. Figure 2 reports the transmission optical image and the reconstructed map of a cell incubated with SeNPs at 15 μg/mL for 24 h, together with the Raman spectra of the different cell regions. Optical micrograph shows the typical elongated shape of fibroblast in health conditions. Black spots can be noticed in the cell and are attributable to nanoparticle aggregates (since microscope magnification does not allow the visualization of individual NPs). These aggregates appear to be located both on the surface and inside the cell, respectively showing well-defined outlines in some cases while presenting blurred ones in others. This fact demonstrates that at these concentrations SeNPs are internalized in HDF cells up to a saturating concentration.

The Raman maps have been reconstructed from the original Raman spectra with the RAMAN Viewer software “area” method (the detailed explanation in Figure S1), without any additional data processing; while the Raman spectra reported in Figure 2b is the result of the data processing described in the Materials and Methods section. Original raw averaged spectra are reported in Figure S2, and it is possible to notice that the CaF_2_ background peak around 320 cm^–1^ is present in all spectra and that none are visible fluorescence effects, especially in the one of SeNPs.

In the range 2800–3000 cm^–1^, the peaks related to CH, CH_2_, and CH_3_ groups are clearly visible.^31,51,52^ Focusing the attention to the shoulder band at 2880 cm^–1^, it has been possible to reconstruct the Raman map of the lipid distribution (Figure 2a, green map).^52^ As expected, lipids are distributed throughout the cytoplasm, composing the membrane of internal organelle, while they are absent within the central nucleus of the cell.^53^ A band related to Amide I group vibrations can be observed at about 1650 cm^–1^.^31,51,52^ This band is typical of proteins and originates from the entire cellular structure, confirming protein distribution throughout the cell, including the nucleus (Figure 2a, red map).^52^ A further confirmation of the correct attribution of the band comes from the possibility of distinguishing the presence of nucleolus structures inside the nucleus (Figure S7, nontreated cell). Indeed, nucleolus are protein-rich regions which can be detected *via* Raman Microspectroscopy.^52,54^

A peak related to CH_2_/CH_3_ vibrations in protein and lipid can be observed at about 1445 cm^–1^.^31,51,55^ The large band between 1200 and 1370 cm^–1^ can be attributed to the convolution of signals related to Amide III vibrations of proteins,^31,51,55^ CH_2_ and CH_3_ vibrations of lipids^51,55^ and adenine and guanine vibrations of DNA.^51,55^ The bands in the range 1070–1145 cm^–1^ are assigned to C–N stretching in protein and C–C in lipids,^55^ while the peak at 1000 cm^–1^ is attributed to phenylalanine amino acid benzene ring vibration.^31,51,52^ The signal around 780 cm^–1^, more pronounced in the nucleus spectra, can be assigned to DNA backbone O–P–O, uracil, cytosine, and thymine (ring breathing) of DNA and RNA.^31,55^ This latter band, together with the small band at 1334 cm^–1^, can be used to qualitatively evaluate the distribution of DNA and RNA within the cell which, as expected, appears to be spread within the nucleus (Figure S8). As previously observed, the two bands at around 500 and 247 cm^–1^ correspond to the vibrations of SeNPs. Interestingly, in the areas analyzed to obtain the average spectrum of the SeNPs dispersed in the cell, the prominent band corresponding to lipid groups (∼2880 cm^–1^) is always present with an intensity comparable to those of the other regions. This observation supports the hypothesis that nanoparticles are taken up by the cell and internalized in lipid-based vesicles, such as endosomes or lysosomes.^34,56,57^ In the map reconstructed from the SeNPs signal at 247 cm^–1^ (Figure 2a, blue map), it is possible to notice a greater number of spots compared to the micrograph image. This confirms the effectiveness of Raman technique to identify small aggregates of selenium nanoparticles, which cannot be observed using optical techniques.

To confirm that SeNPs are penetrating the membrane rather than attaching to it, confocal Raman microspectroscopy was employed on HDF cells, following 24 h exposure to 15 μg/mL of SeNPs. To optimize data acquisition and minimize 3D image reconstruction times, the cells were analyzed with a reduced laser exposure time of 5 s per line (compared to the 10 s acquisition for 2D maps). This optimized approach allowed acquisition times to be reduced to approximately 1 h for a single 3D map while mitigating potential laser-induced cellular deterioration during measurement. Nevertheless, fewer nanoparticles could be resolved due to the lower signal-to-noise ratio. However, if higher resolution is required, longer acquisition times or increased laser power can be employed, although calibration may be necessary, as nanoparticles can be altered under high laser powers.

**Figure 3:**
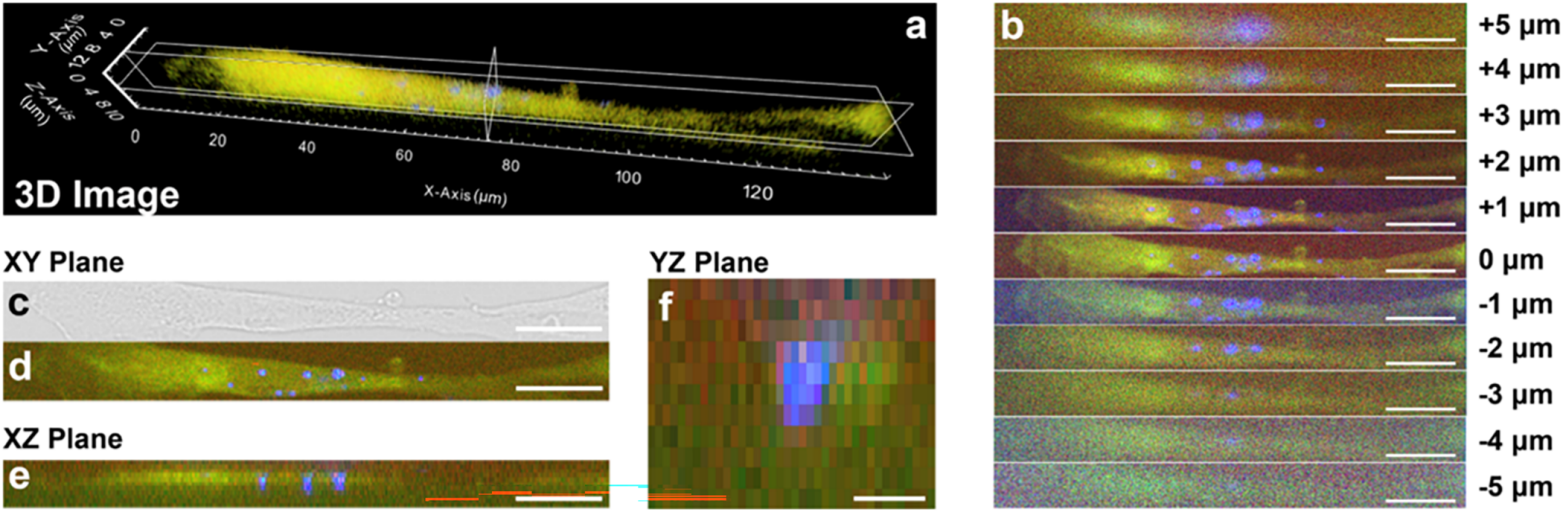
(a) Confocal 3D Raman image of an HDF cell exposed to 15 μg/mL of SeNPs for 24 h, reconstructed from (b) 2D maps collected every 1 μm step in the *z*-direction. (c) Optical micrograph and Raman images along the (d) *XY*, (e) *XZ*, and (f) *YZ* planes corresponding to the center of the cell (b–e scale bar: 20 μm; *f* scale bar: 4 μm). Colors of the images correspond to the merging of the signals at ∼2880 cm^–1^ (lipids, green), ∼1650 cm^–1^ (proteins, red), and ∼247 cm^–1^ (SeNPs, blu).

Figure 3 and Movie S1 display the 3D image of the cell obtained by combining the 2D Raman maps acquired at different focusing depths, assigning “0 μm” as the cell central plane. Also in this case, HDF cells display the elongated shape typical of fibroblasts in health conditions. However, no sign of superficial or internal NPs could be detected from the optical transmission image alone. On the contrary, SeNPs could be localized through the reconstruction of Raman maps. Several NPs can be detected in the peripheral area of the nucleus, with a single spot apparently visible in the internal part. By observation of the cell from the *XZ* and *YZ* planes, it is possible to notice that the SeNPs signals can be detected in the central areas of the images. This confirms the internalization of SeNPs inside HDF cells and further supports the Confocal Raman Microspectroscopy technique as a simple and effective method for their intracellular detection. The high specificity of the Raman signal of SeNPs, combined with Confocal Raman microscopy, could also be leveraged for future quantitative analyses of the internalized nanoparticles. A potential approach for quantification may involve correlating the number of SeNPs within the Raman laser spot with the corresponding Raman signal intensity, enabling a precise estimation of intracellular nanoparticle concentration. However, this method would require rigorous calibration, which was beyond the scope of the present study.

**Figure 4:**
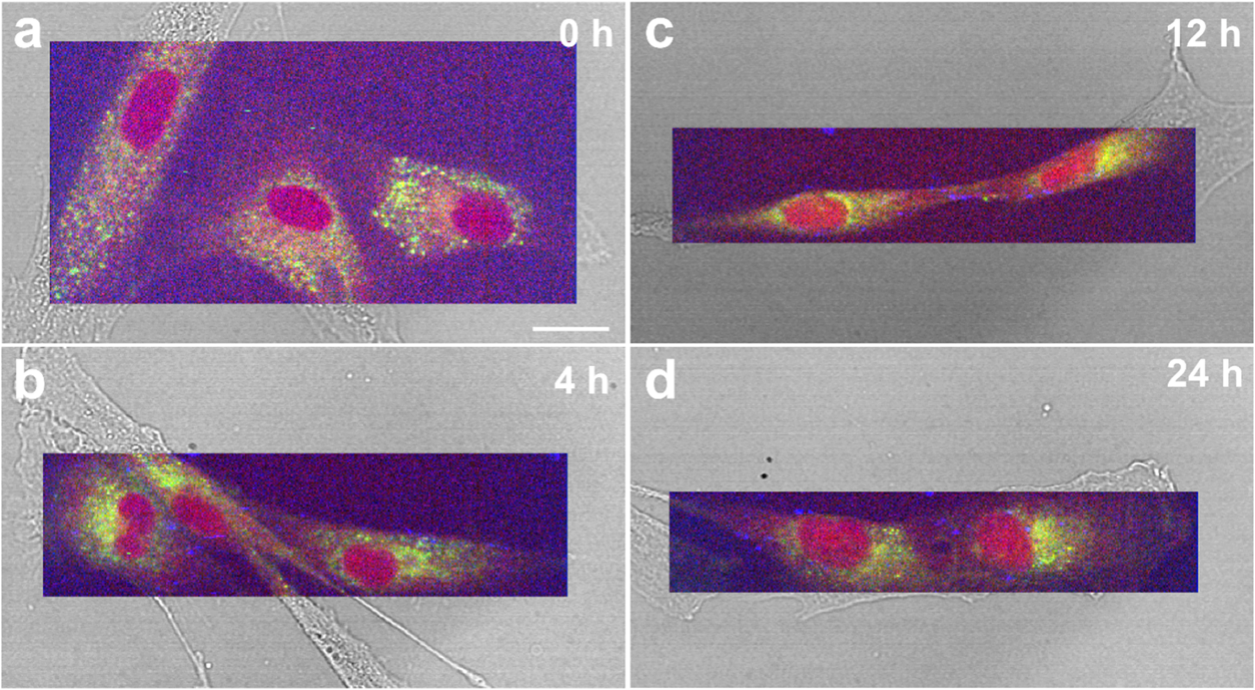
Raman images of HDF cells exposed to 1.5 μg/mL of SeNPs for (a) 0, (b) 4, (c) 12, and (d) 24 h. Colors of the images correspond to the merging of the signals at ∼2880 cm^–1^ (lipids, green), ∼1650 cm^–1^ (proteins, red), and ∼247 cm^–1^ (SeNPs, blu). Scale bar: 20 μm.

Despite the promising results obtained, the concentration of 15 μg/mL appears to be excessively high for possible biomedical applications, as evidenced by cytotoxicity tests over 72 h and by the presence of cells containing a very large number of nanoparticles and aggregates within them (Figure S9), which could potentially induce signal alterations or unexpected cellular behaviors. Consequently, we decided to perform uptake dynamic and localization studies at different time points (0, 4, 12, and 24 h) at a concentration 10 times lower (1.5 μg/mL) to ensure nonharmful conditions (Figure 4). In this way, even if the SeNPs would dissolve, the released toxic Se ions would not cause detrimental action, as they would be in a low amount and could more easily be reused by the cells or diffuse. As expected, before the addition of SeNPs (Figures 4a and S10), no peak at 247 cm^–1^ can be detected, thus demonstrating that only SeNPs contribute to this signal. After 4 h of exposure to SeNPs, few particles can be identified inside the cell membranes, proving that an initial uptake already occurs within the first few hours of exposure (Figure 4b). Interestingly, the cell depicted on the left in Figure 4b was about to complete its mitotic process, demonstrating that the presence of SeNPs at this concentration does not hinder normal cellular processes, allowing the cells to correctly divide. The amount of nanoparticles within the cells does not seem to increase over time, remaining approximately constant during 24 h (Figure 4d). Nevertheless, this fast uptake highlights their potential as a valuable biomedical tool, as their rapid internalization can facilitate timely therapeutic or diagnostic applications. In addition, at each time point, cells showed no signs of anomalous morphology, once again supporting the hypothesis that SeNPs are not hazardous at this concentration. Eventually, the lack of 247 cm^–1^ signals from inside the nuclei confirms that SeNPs cannot penetrate the nuclear membrane, at least at the investigated concentrations. In fact, in the absence of active transport mechanisms, nanoparticles generally need to be smaller than about 10 nm to pass through the nuclear pores for passive diffusion.^58^ On the other hand, nanoparticles up to ∼100 nm can penetrate the cell membrane, with a size of 10–50 nm being optimal for endocytosis.^52,59^ Our result supports the hypothesis that SeNPs cannot interact and hinder genomic-related cellular functions, consequently making them potentially less harmful.

## Conclusions

In this study, we have demonstrated for the first time the efficacy of Confocal Raman Microspectroscopy as a potent analytical tool for investigating the intracellular localization of SeNPs. Our research highlights that SeNPs can be easily and cost-effectively synthesized with a homogeneous distribution. Furthermore, these nanoparticles exhibited good biocompatibility with healthy human cells, underscoring their potential for a wide array of biomedical applications. One of the most compelling advantages of SeNPs is their specific Raman signal, which does not overlap with the spectral fingerprint of cellular components. 2D and 3D Raman maps on cells treated with SeNPs have been obtained, confirming their penetration inside the cellular membrane even at low concentrations (1.5 μg/mL) and after a short period of time (4 h). The ability to monitor these nanoparticles without additional markers simplifies the imaging process and reduces potential interference from labeling agents. Moreover, the potential for functionalizing SeNPs with peptides or other bioactive molecules could further mitigate any residual toxicity and provide the nanoparticles with additional desirable properties. This functionalization could lead to the development of highly specialized and targeted therapeutic agents. Overall, the combination of SeNPs’ biocompatibility, specific and nonoverlapping Raman signal, and their facile synthesis makes them a highly promising platform for future biomedical and bioimaging applications.

## Supporting Information Available

Magnification of raw averaged Raman spectra and description of map reconstruction method; Raman map showing the areas from which raw averaged Raman spectra have been acquired; SEM image and SEM and DLS distributions of SeNPs after 10 months in fridge; XRD diffractogram, TEM images, diffraction pattern, and raw Raman spectra of SeNPs; sedimentation velocity distribution for four SeNPs sizes; fluorescence spectra of SeNPs; Raman image of untreated cell showing nucleolus; additional Raman images of DNA and RNA signals; Raman image of HDF cells with large number of SeNPs; and representative Raman spectra of a nontreated cell (PDF)
Rotating 3D image of the cell reported in Figure 3 (MP4)

## Abbreviations

SeNPs: selenium nanoparticles
HDF: human dermal fibroblast
AuNPs: gold nanoparticles
AgNPs: silver nanoparticles
