# Supplementary material for: Mapping Selenium Nanoparticles Distribution Inside Cells through Confocal Raman Microspectroscopy
Source: ACS Appl Mater Interfaces. 2025 Mar 18;17(12):18124–33. doi: 10.1021/acsami.5c00380 (PMC11956006; doi:10.1021/acsami.5c00380)
Supplement: Supplementary file 1 — am5c00380_si_001.pdf [file am5c00380_si_001.pdf]

SUPPORTING INFORMATION

Mapping Selenium Nanoparticles Distribution Inside Cells through Confocal Raman Microspectroscopy

*Davide Redolfi-Bristol,[*1,2] Kenta Yamamoto,[3] Wenliang Zhu,[1] Osam Mazda,[3] Pietro Riello[2], Elia Marin,[*4,5,6] Giuseppe Pezzotti,[2,3,7,8,9,10]*

[1] Ceramic Physics Laboratory, Kyoto Institute of Technology, Sakyo-ku, Matsugasaki, 606-8585, Kyoto, Japan

[2] Dipartimento di Scienze Molecolari e Nanosistemi, Università Ca' Foscari di Venezia, Via Torino 155, 30172 Venezia, Italia.

[3] Department of Immunology, Graduate School of Medical Science, Kyoto Prefectural University of Medicine, 465 Kajii-cho, Kamigyo-ku, Kyoto 602-8566, Japan

[4] Biomaterials Engineering Laboratory, Kyoto Institute of Technology, Sakyo-ku, Matsugasaki, 606-8585, Kyoto, Japan

[5] Department Polytechnic of Engineering and Architecture, University of Udine, 33100, Udine, Italy

[6] Biomedical Research Center, Kyoto Institute of Technology, Sakyo-ku, Matsugasaki, Kyoto 606-8585, Japan

[7] Biomedical Engineering Center, Kansai Medical University, 1-9-11 Shinmachi, Hirakata, Osaka 573-1191, Japan

[8] Department of Dental Medicine, Graduate School of Medical Science, Kyoto Prefectural University of Medicine, 465 Kajii-cho, Kamigyo-ku, Kyoto 602-8566, Japan

[9] Department of Orthopedic Surgery, Tokyo Medical University, 6-7-1 Nishi-Shinjuku, Shinjuku-ku, 160-0023 Tokyo, Japan

[10] Department of Applied Science and Technology, Politecnico di Torino, Corso Duca degli Abruzzi 24, 10129 Torino, Italy

* E-mail: davide.redolfi@unive.it
* E-mail: elia-marin@kit.ac.jp

TABLE OF CONTENTS

FIGURE S1. Magnification of a raw averaged Raman spectra and description of map reconstruction method……S2

FIGURE S2. Raman map showing the areas from which raw averaged Raman spectra has been acquired.……..S3

FIGURE S3. SEM image and SEM and DLS distributions of SeNPs after 10 months in fridge…………...……..S4

FIGURE S4. XRD diffractogram, TEM images, diffraction pattern, and raw Raman spectra of SeNPs…..……S4

FIGURE S5. Sedimentation velocity distribution for four SeNPs sizes.…………………………………………S5

FIGURE S6. Fluorescence spectra of SeNPs………………………. ……………………………………………S6

FIGURE S7. Raman image of untreated cell showing nucleolus……..…………………………………………S6

FIGURE S8. Additional Raman images of DNA and RNA signals….. …………………………………………S7

FIGURE S9. Raman image of HDF cells with large number of SeNPs …………………………………………S7

FIGURE S10. Representative Raman spectra of a non-treated cell ………………………………………………S8

MOVIE S1. Rotating 3D image of the cell reported in Figure 3.…………………………………………………S8

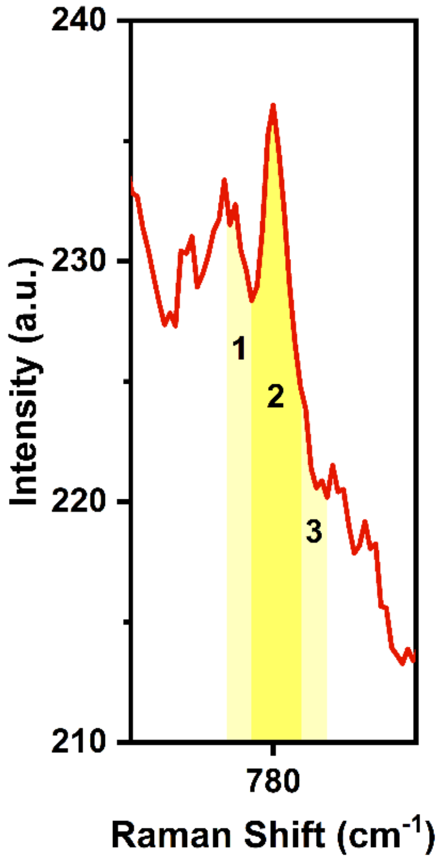

$$(1) \quad \textbf{\textit{Pixel Color Intensity}} = \textbf{\textit{Area}}_2 - (\textbf{\textit{Area}}_1 + \textbf{\textit{Area}}_3)$$

Figure S1. Magnification of a raw averaged Raman spectra. The color intensity of each pixel of the Raman maps is obtained using formula (1). Here, $Area_2$ represents the integrated area under the curve highlighted by the darker yellow region, while $Area_1$ and $Area_3$ correspond to the integrated areas under the curve delineated by the lighter yellow regions.

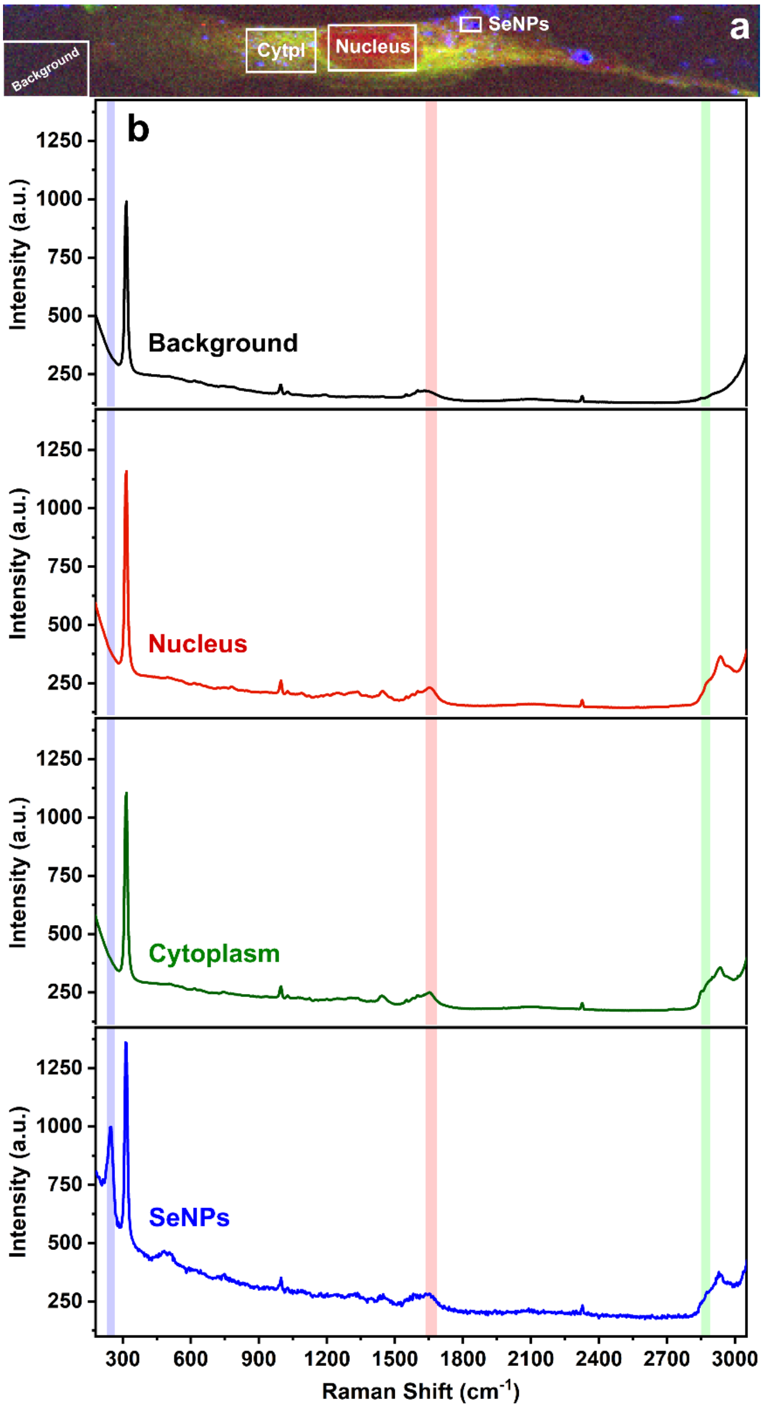

Figure S2. (a) Color merged Raman image (same of Figure 2) reporting the rectangular areas from which the raw averaged spectra of the background, cytoplasm, nucleus and internalized SeNPs have been obtained and (b) corresponding raw average Raman spectra of the different areas. It is possible to notice that the CaF_2 background peak around 320 cm^{-1} is present in all spectra and that in none is visible fluorescence effects, especially not in the one of SeNPs

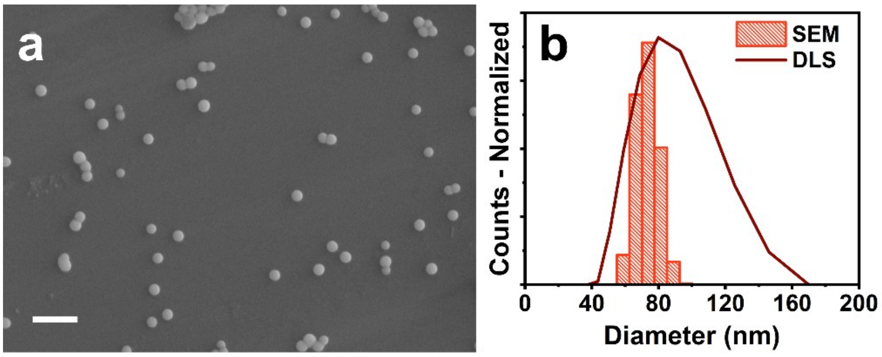

Figure S3. (a) SEM image (scale bar: 300 nm) and (b) SEM and DLS distributions of SeNPs kept in fridge at 4 °C for 10 months.

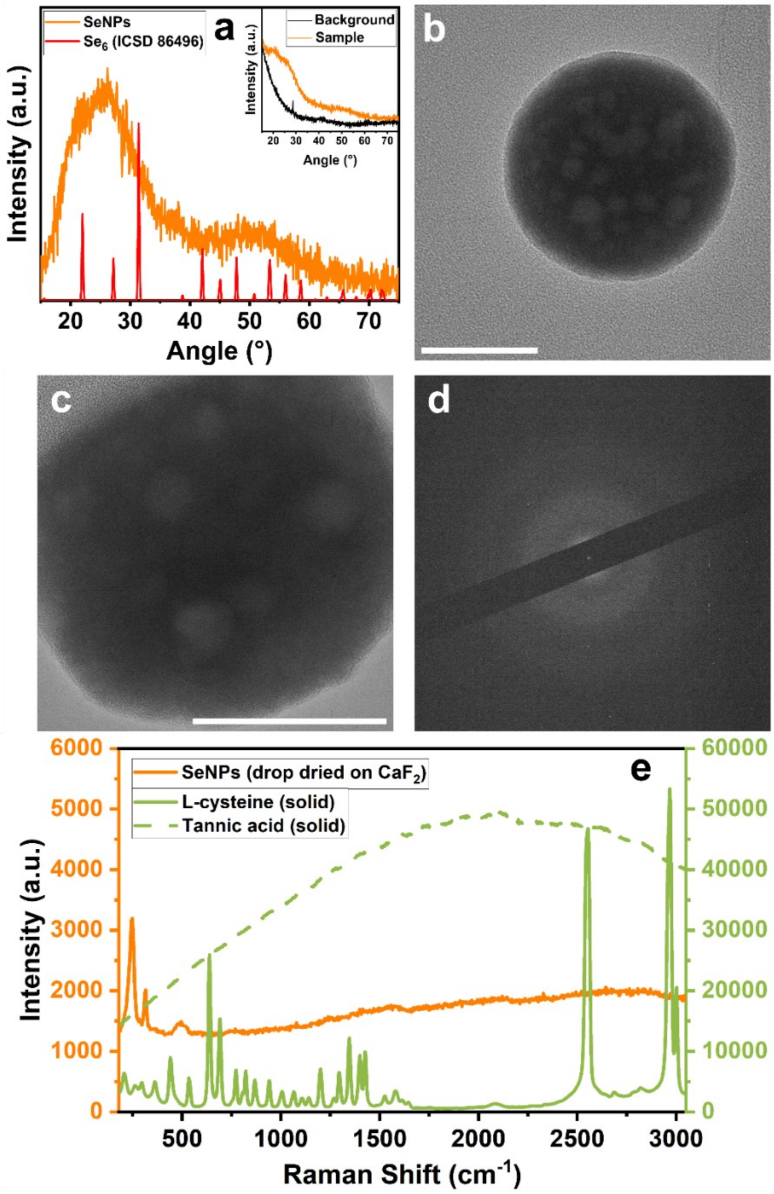

Figure S4. (a) XRD diffractogram of SeNPs obtained by subtracting the background signal from the diffractogram of the SeNPs deposited and dried on a "zero background"; (b,c) TEM images (scale

bar: 50 nm) and (d) diffraction pattern of SeNPs; (e) Raw Raman spectra of the drop dried of SeNPs solution, and solid powder of L-cysteine and Tannic acid; left scale (in orange) refers to the SeNPs' signal intensity, while right scale (in green) refers to pure L-cysteine and tannic acid signal intensity

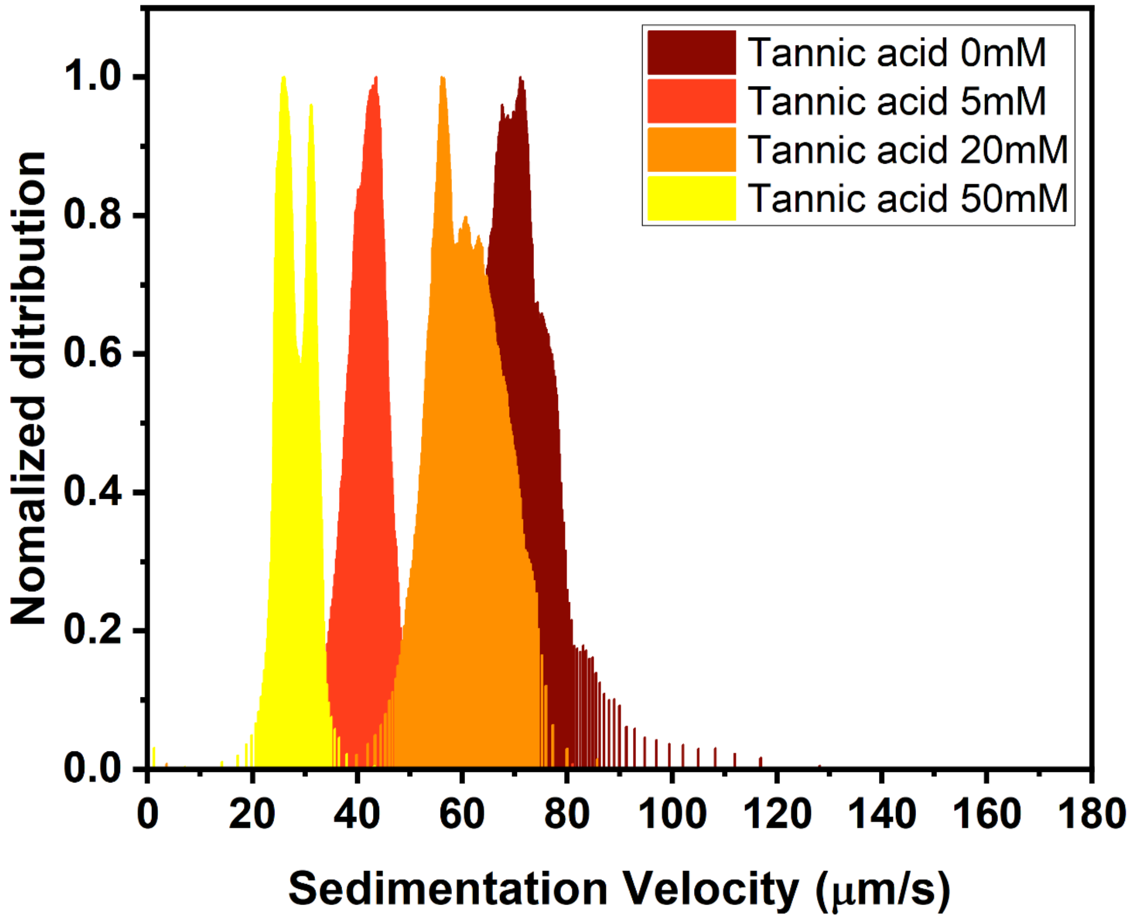

Figure S5. Sedimentation velocity distribution measured by analytical centrifuge for four different sizes of SeNPs synthesized by varying the concentration of tannic acid in solution

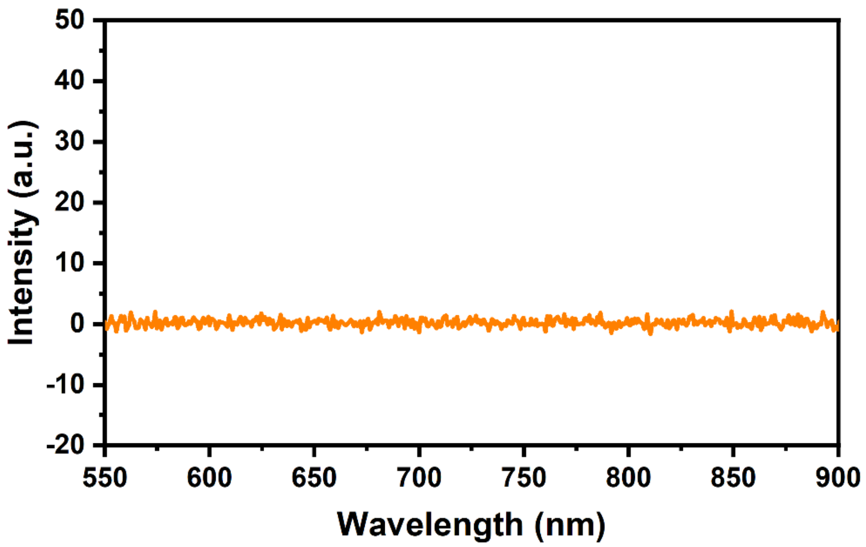

Figure S6. Fluorescence spectra of the colloidal solution of SeNPs excited by an LED source at 510 nm and in a detection window of 550-900 nm

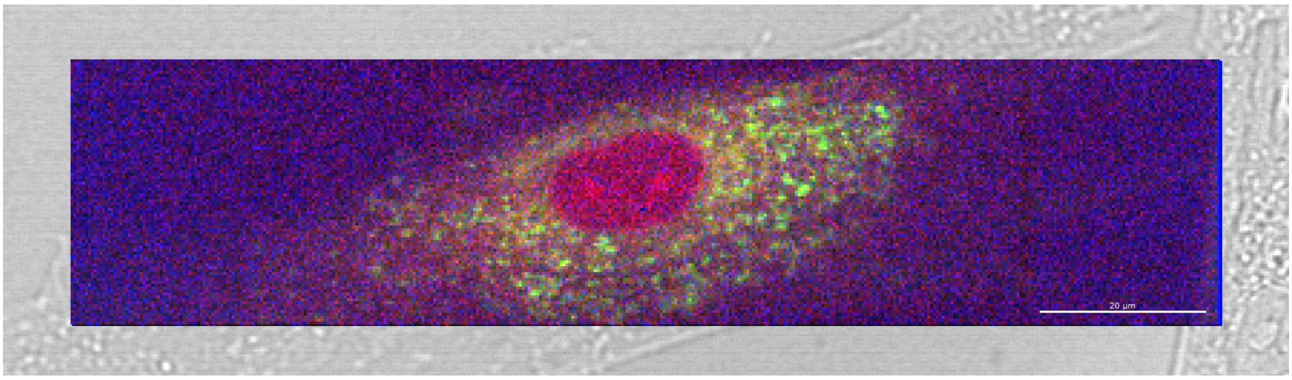

Figure S7. Raman image obtained from merging of green, red and blue maps of non-treated HDF cells (scale bar: 20 µm). Inside the nucleus it is possible to notice two spots of a more intense red color corresponding to the nucleoli structures

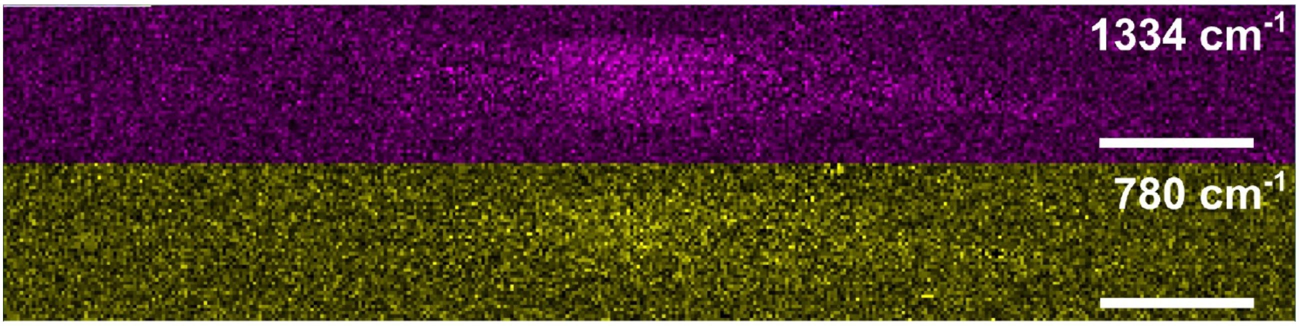

Figure S8. Raman images obtained from signals at ~1334 cm^{-1} and ~780 cm^{-1} (DNA and RNA) of HDF cells incubated with SeNPs at 15 µg/mL for 24 h. Scale bar: 20 µm

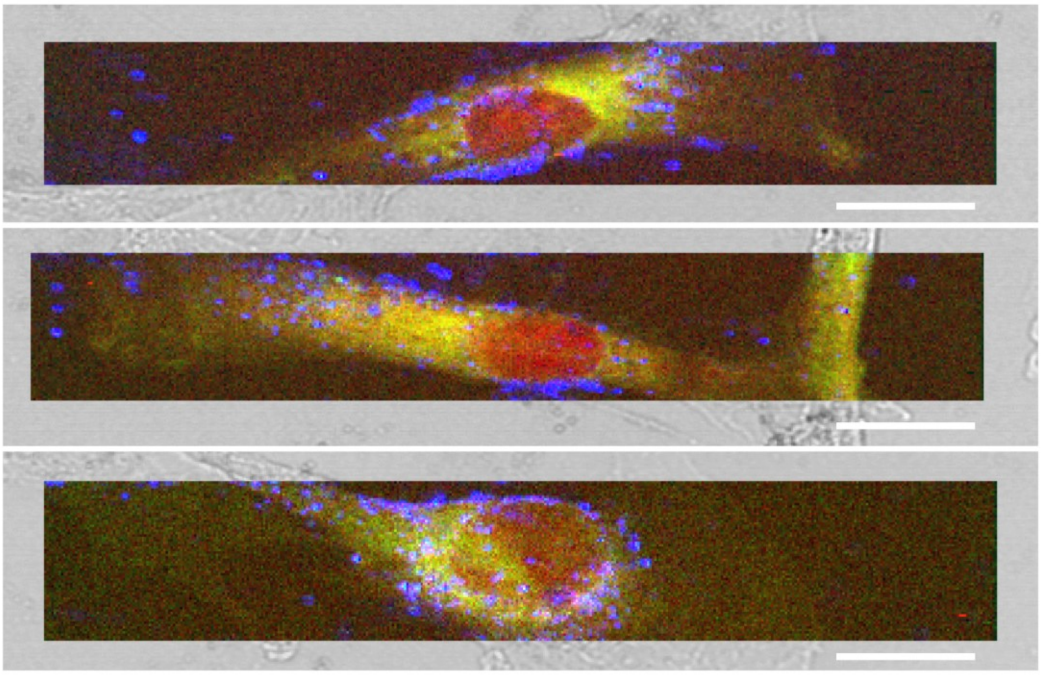

Figure S9. Raman images obtained from merging of green, red and blue maps of HDF cells incubated with SeNPs at 15 µg/mL for 24 hours (scale bar: 20 µm). Inside cells and in the extracellular area it is possible to notice a large number of SeNPs

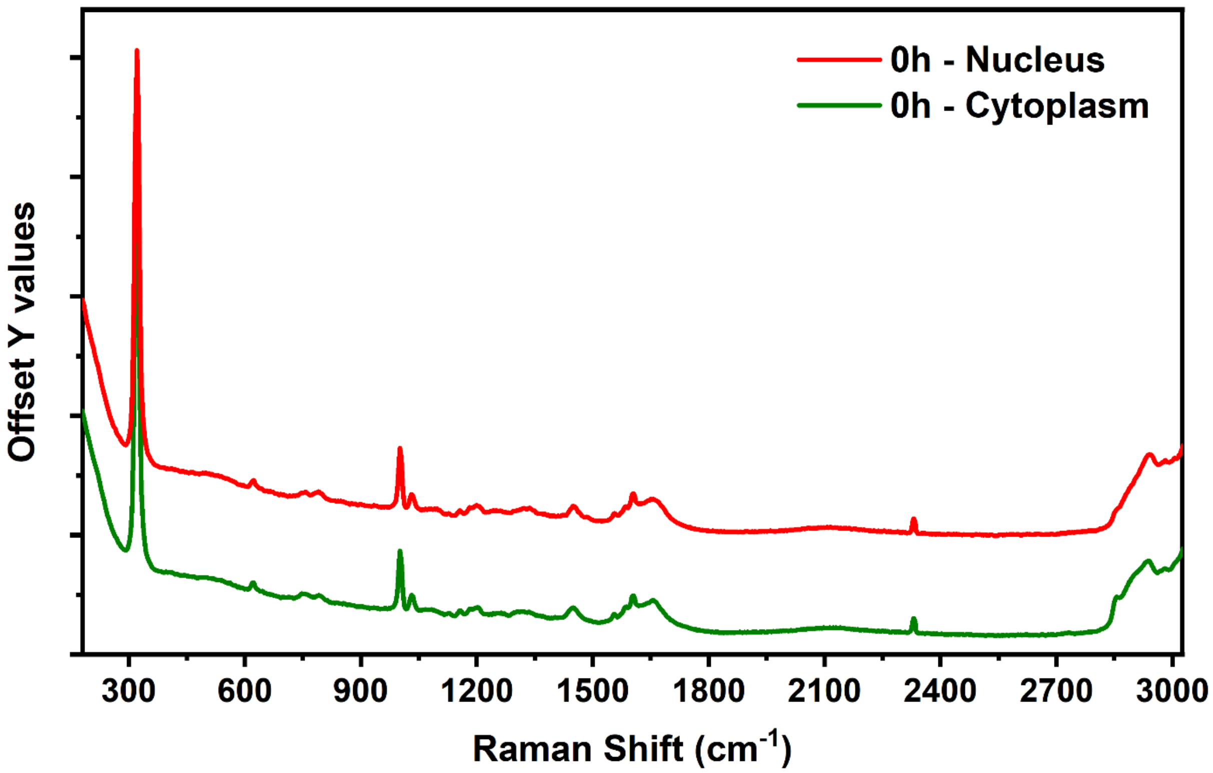

Figure S10. Representative Raman spectra of cytoplasm and nucleus of a non-treated cell. It is possible to notice the absence of any peak at 247 cm^{-1}

MOVIE S1. Rotating 3D image of the cell reported in Figure 3
